# Clinical and electroencephalographic particularities of children and adolescents with behavioral disorders

**DOI:** 10.1192/j.eurpsy.2024.923

**Published:** 2024-08-27

**Authors:** R. Bouchech, A. Fki, I. Kammoun, K. Masmoudi

**Affiliations:** ^1^Functional explorations department, Habib Bourguiba hospital, Sfax; ^2^Occupational medicine department, Sahloul hospital, Sousse, Tunisia

## Abstract

**Introduction:**

Behavioral disorders are a frequent reason for consultation in child psychiatry. Children and adolescents with epilepsy are at risk of behavioral disorders that can affect their quality of life.

**Objectives:**

The aim of this study was to investigate the electroencephalographic aspects of children with behavioral disorders and to determine the prevalence of comorbidity with epilepsy.

**Methods:**

This was a retrospective descriptive study conducted from January 2019 to May 2022. We included all children and adolescents referred to the functional explorations department at Habib Bourguiba hospital, Tunisia for Electroencephalogram (EEG) as part of a workup to explore a behavioral disorder.

**Results:**

A total of 117 patients were included in the study. The mean age was 14 ±4.2 years. The sex ratio was 1.29. Agitation was reported in 66.7% of patients. One case of attempted suicide was noted. Among these patients, 29.9% reported audiovisual hallucinations. Concentration difficulties were associated with 59% of cases. Ten patients had a history of epileptic seizures. Of the 117 EEGs performed, 59.8% were pathological. The abnormalities observed were paroxysms in 67.1% of cases and focal slowing in 25.7%. Five patients had a rapid rhythm on the EEG. An absence-type electro-clinical seizure was recorded in one patient. Patients with visual hallucinations had epileptiform abnormalities of occipital location in 41.7% of cases, and slow waves of anterior location in 50% of cases. Patients with auditory hallucinations had parietal epileptiform abnormalities in 89% of cases.

**Conclusions:**

Ictal and interictal manifestations seem to play a part in the genesis of behavioral disorders in children and adolescents. An EEG would therefore be preferable in this age group, for better management.

**Disclosure of Interest:**

None Declared

